# Nasal vaccine delivery attenuates brain pathology and cognitive impairment in tauopathy model mice

**DOI:** 10.1038/s41541-020-0172-y

**Published:** 2020-03-25

**Authors:** Hiroki Takeuchi, Keiko Imamura, Bin Ji, Kayoko Tsukita, Takako Enami, Keizo Takao, Tsuyoshi Miyakawa, Masato Hasegawa, Naruhiko Sahara, Nobuhisa Iwata, Makoto Inoue, Hideo Hara, Takeshi Tabira, Maiko Ono, John Q. Trojanowski, Virginia M.-Y. Lee, Ryosuke Takahashi, Tetsuya Suhara, Makoto Higuchi, Haruhisa Inoue

**Affiliations:** 1 Center for iPS Cell Research and Application (CiRA), Kyoto University, 53 Shogoin, Kawahara-cho, Sakyo-ku, Kyoto, Kyoto, 606-8507 Japan; 2 iPSC-based Drug Discovery and Development Team, RIKEN BioResource Research Center (BRC), Kyoto, Japan; 3 Medical-risk Avoidance based on iPS Cells Team, RIKEN Center for Advanced Intelligence Project (AIP), Kyoto, 606-8507 Japan; 4 Department of Functional Brain Imaging, National Institute of Radiological Sciences, National Institutes for Quantum and Radiological Science and Technology, 4-9-1 Anagawa, Inage-ku, Chiba, Chiba, 263-8555 Japan; 5 Graduate School of Innovative Life Science, University of Toyama, Toyama, 930-0194 Japan; 6 Life Science Research Center, University of Toyama, Toyama, 930-0194 Japan; 7 Genetic Engineering and Functional Genomics Group, Frontier Technology Center, Graduate School of Medicine, Kyoto University, Yoshida-Konoe-cho, Sakyo-ku, Kyoto, 606-8501 Japan; 8 Department of Dementia and Higher Brain Function, Tokyo Metropolitan Institute of Medical Science, 2-1-6 Kamikitazawa, Setagaya-ku, Tokyo, 156-8506 Japan; 9 Department of Genome-based Drug Discovery, Graduate School of Biomedical Sciences, Nagasaki University, Nagasaki, 852-8521 Japan; 10 Sumitomo Dainippon Pharma Co., Ltd., 13-1, Kyobashi 1-Chome, Chuo-ku, Tokyo, 104-8356 Japan; 11 Department of Internal Medicine, Saga University Faculty of Medicine (Saga Medical School), 5-1-1 Nabeshima, Saga, 849-8501 Japan; 12 Department of Diagnosis, Prevention and Treatment of Dementia, Faculty of Medicine, Juntendo University, 2-11-5 Hongo, Bunkyo-ku, Tokyo, 113-0333 Japan; 13 Center for Neurodegenerative Disease Research, University of Pennsylvania Perelman School of Medicine, 3rd Floor HUP-Maloney, 36th and Spruce Streets, Philadelphia, PA 19104 USA; 14 Department of Neurology, Graduate School of Medicine, Kyoto University, 54 Shogoin, Kawahara-cho, Sakyo-ku, Kyoto, Kyoto, 606-8507 Japan

**Keywords:** Immunology, Diseases

## Abstract

Pathological aggregates of tau proteins accumulate in the brains of neurodegenerative tauopathies including Alzheimer’s disease and frontotemporal lobar degeneration (FTLD-tau). Although immunotherapies of these disorders against tau are emerging, it is unknown whether nasal delivery, which offers many benefits over traditional approaches to vaccine administration, is effective or not for tauopathy. Here, we developed vaccination against a secreted form of pathological tau linked to FTLD-tau using a Sendai virus (SeV) vector infectious to host nasal mucosa, a key part of the immune system. Tau vaccines given as nasal drops induced tissue tau-immunoreactive antibody production and ameliorated cognitive impairment in FTLD-tau model mice. In vivo imaging and postmortem neuropathological assays demonstrated the suppression of phosphorylated tau accumulation, neurotoxic gliosis, and neuronal loss in the hippocampus of immunized mice. These findings suggest that nasal vaccine delivery may provide a therapeutic opportunity for a broad range of populations with human tauopathy.

## Introduction

Pathological species of microtubule-associated protein tau (MAPT)^1^ accumulate in the brain of Alzheimer’s disease (AD) and diverse non-AD neurodegenerative disorders collectively referred to as tauopathies, including frontotemporal lobar degeneration (FTLD) characterized by tau deposition (FTLD-tau)^2^. Unequivocal evidence for mechanistic links between tau abnormalities and neurodegeneration was provided by discoveries of tau gene mutations in familial forms of FTLD-tau termed frontotemporal dementia with parkinsonism linked to chromosome 17 (FTDP-17). Current research has indicated that the inhibition of tau misfolding exerts a therapeutic effect in transgenic mice expressing FTDP-17 mutant tau^3–5^, although neither pharmacologic nor non-pharmacologic intervention targeting tau pathologies has been clinically successful. Moreover, tau is released from neurons into extracellular space^6,7^, raising the importance of clearing extracellular tau by immunotherapy including anti-tau vaccination, which has emerged as a promising approach to tauopathies^8,9^. Several different lines of studies have demonstrated the efficacy of tau vaccines in transgenic mouse models of tauopathies^10–13^. Vaccine-derived antibodies against tau proteins may interact with misfolded tau species by entering neurons^13,14^, may block extracellular seeds of tau aggregates^11^, or may enhance microglial clearance of intercellularly transmitting tau^15^, although the induction of neuroinflammation by vaccinations has been reported^16^.

Accumulated evidence in basic research supports the idea that immunotherapy against tau may be a promising therapeutic approach for tauopathy. However, nasal vaccination has not been tested against tauopathy, even though it offers many benefits over traditional vaccine approaches^17^, including ease of administration without needles, to vaccine administration. Here, we developed nasal tau vaccination using Sendai virus (SeV) vector. SeV is a murine parainfluenza virus type 1 which has a negative-sense single-stranded RNA^18^, and has been used for the delivery of foreign genes to host cells^19^. It has the potential to enter broad species and cell types by using sialic acid on the host cell surface^20^. The replication and transcription of our SeV vector occur and continue for about a month after administration in the cytoplasm of epithelial cells in the respiratory system^21,22^, and its gene never integrates into the host DNA^23^. SeV also naturally replicates in respiratory mucosa, and accordingly a preferential route for SeV vector administration is intranasal, which could offer the advantage of the induction of mucosal immune responses^24–26^. Clinical trials using SeV vectors for gene therapy or vaccination have been conducted, and they showed the safety of these treatments in humans^27–29^. We constructed a SeV vector, which resulted in secreted tau being released into the extracellular space as an antigen, and we investigated the therapeutic effects of the vaccine in a murine model of tauopathy through imaging and behavioral tasks.

## Results

### Generation of nasal vaccine against tau

**Fig. 1:**
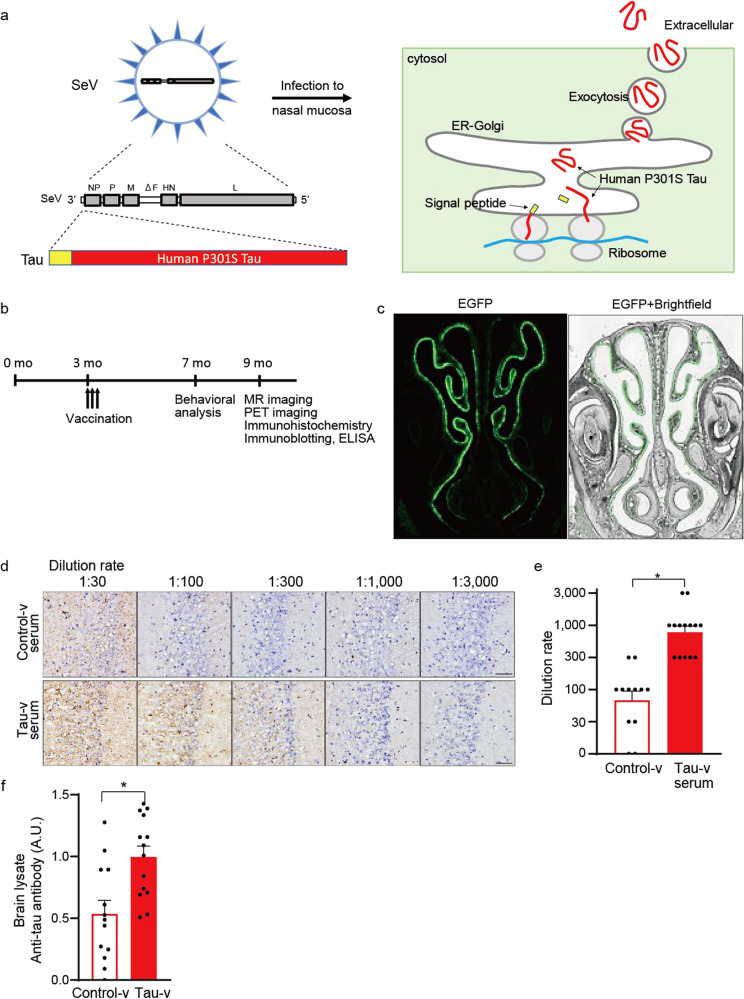
Generation of nasal vaccine against tau. **a** Schematic diagram of the nasal vaccination of immunogen protein using SeV vector. The vector encodes the signal peptide combined with human P301S mutant tau protein, and the vaccine is delivered to the extracellular space. **b** Time course of the experiments. **c** EGFP expression in the nasal mucosa of mice 1 week after the administration of SeV vector encoding EGFP. **d** Vaccine-enhanced generation of tissue tau-specific antibodies. Immunohistochemical detection of tissue tau with serum of FTLD-tau mice. Serum from tau-v-treated FTLD-tau mice reacted with putative intraneuronal tau deposits in the hippocampal CA3 sector of an untreated FTLD-tau mouse more potently than did control serum. Scale bars: 50 µm. **e** Quantification of tissue tau reactivity. Increased levels of tissue tau-reactive antibodies in tau-v-treated FTLD-tau mice were verified by quantitative ranking analysis of the dilution rate in immunolabeling. *p < 0.005 by *t*-test (control-v-treated FTLD-tau mice, *n* = 12; tau-v-treated FTLD-tau mice, *n* = 14). **f** Quantification of anti-human tau antibody detected in brain lysates. Brain lysates of mice treated with tau-v showed an increase in anti-human tau antibody compared with those of mice treated with control-v. *p < 0.005 by *t*-test (control-v-treated FTLD-tau mice, *n* = 13; tau-v-treated FTLD-tau mice, *n* = 14). All blots were derived from the same experiment and were processed in parallel.

It has been documented that mutant tau proteins provoke the generation of misfolded tau, leading to the deposition of tau fibrils and tau-induced neurotoxicity in FTDP-17 patients and transgenic mouse models^30,31^ and giving a rationale for immunotherapeutically targeting pathological tau by vaccination. For tau vaccination (tau-v), we constructed a SeV vector carrying the cDNA of human tau isoform with one N-terminal insert and four repeat domains (1N4R) with the P301S mutation^32^ as an immunogen fused to a signal peptide for directing gene products to a secretory pathway^33^ (Fig. 1a, Supplementary Fig. 1a). Before vaccination to mice, we confirmed that enhanced secretion of mutant tau into the extracellular matrix by incorporation of this signal peptide by immunoprecipitating tau-v-infected cell culture media (Supplementary Fig. 1b), which led to us detecting misfolded tau protein in the culture medium of tau-v-infected cells (Supplementary Fig. 1c). SeV vectors were administered to mice intranasally, and the effects of the vaccination were evaluated (Fig. 1b). Efficient yields of an exogenous gene product in olfactory mucosa following nasal administration to mice were demonstrated with the use of SeV vector encoding enhanced green fluorescent protein (EGFP) (Fig. 1c). In order to explore immunoreactions following tau-v inoculation, we analyzed anti-tau antibodies generated in mouse serum. The production of antibodies reactive to tau deposits in the hippocampus of PS19 mice (FTLD-tau mice) was confirmed by treatment with serum from mice treated with tau-v relative to control-v (Fig. 1d, e). In addition, an increase of anti-tau antibodies in the brain of mice treated with tau-v was detected (Fig. 1f, Supplementary Fig. 1d). These antibodies are IgG antibodies, but no IgA antibodies were detected.

### Evaluation of effects of tau vaccine on FTLD-tau mice using MRI and PET imaging

**Fig. 2:**
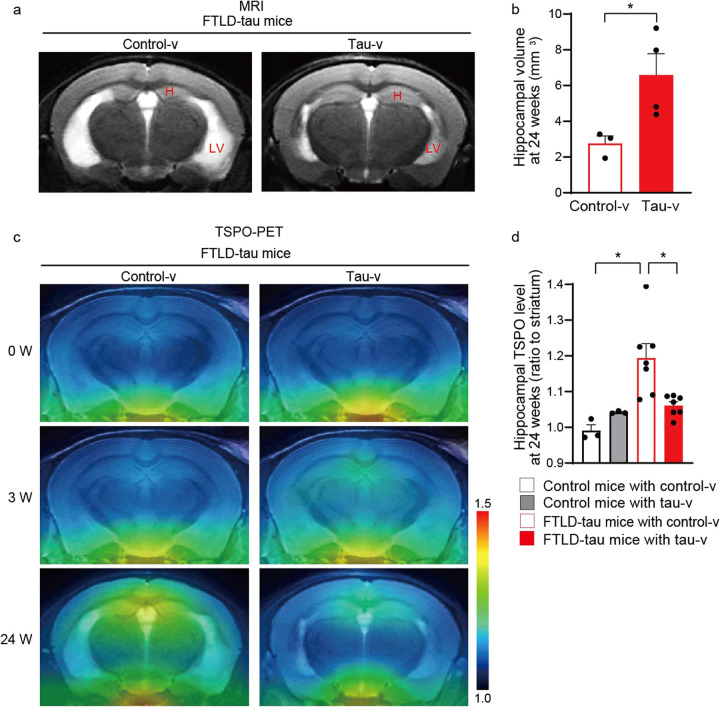
Evaluation of effects of nasal vaccination by neuroimaging. **a** Coronal T2-weighted MR images of FTLD-tau mouse brains showing the anterior hippocampus (H) and lateral ventricle (LV) at 24 weeks after vaccination. **b** Hippocampal volumes in FTLD-tau mice measured by MRI at 24 weeks after vaccination are shown. *p < 0.05 by *t*-test (control-v-treated FTLD-tau mice, *n* = 3; tau-v-treated FTLD-tau mice, *n* = 4). **c** Coronal TSPO-PET images of FTLD-tau mouse brains containing the anterior hippocampus at 0, 3, and 24 weeks after vaccination. PET images were generated by averaging the dynamic scan data at 30–60 min after the injection of [^11^C]Ac5216. The color scale indicates a ratio of the radioactivity retention to the striatum. **d** TSPO levels in control and FTLD-tau mice at 24 weeks after vaccination. There was a significant main effect of genotype [*F*(1,16) = 11.17, *p* < 0.005] and a significant interaction between genotype and treatment [*F*(1,16) = 7.677, *p* < 0.05] by two-way ANOVA. *p < 0.05 by Bonferroni’s post-hoc test (control-v-treated control mice, *n* = 3; tau-v-treated control mice, *n* = 3; control-v-treated FTLD-tau mice, *n* = 7; tau-v-treated FTLD-tau mice, *n* = 7). All blots were derived from the same experiment and were processed in parallel.

FTLD-tau mice were reported to develop a progressive accumulation of misfolded tau, synaptic loss and inflammatory gliosis^30^, leading to the manifestation of phenotypic abnormalities at around 6 months of age^34^ and neuronal loss represented by magnetic resonance imaging (MRI)-detectable hippocampal atrophy at 9–12 months^30^. FTLD-tau mice received nasal inoculation of tau-v or control-v at 3 months of age, and then were evaluated by MRI at 9 months. Volumetric analyses of the MRI data demonstrated an alleviation of hippocampal atrophy by tau-v (Fig. 2a, b), indicating that tau-v suppressed neuronal loss.

These FTLD-tau mice were also subjected to longitudinal positron emission tomographic (PET) scans to assess the time course of the neuroinflammatory changes. In vivo PET imaging with [^11^C]Ac5216, a radioligand for 18-kDa translocator protein (TSPO), a marker for detrimental microgliosis^35^, indicated that FTLD-tau mice with control-v began to show an elevated level of TSPO in the hippocampus at 6 months of age, and this became even more pronounced at 9 months. This relatively late change in TSPO-PET was attributable to robust microgliosis, as has been previously documented^35,36^, and was notably suppressed by tau-v treatment (Fig. 2c, d, Supplementary Fig. 2a), although a modest, insignificant rise of TSPO-PET signals in the hippocampus was transiently observed at 3 weeks after immunization with tau-v (Supplementary Fig. 2b). A PET radioligand for fibrillary tau inclusions, [^11^C]PBB3^37^, is capable of visualizing densely packed tau aggregates in the brainstem of FTLD-tau mice, although [^11^C]PBB3-PET does not detect neurotoxic pre-tangles, the major tau pathology in the hippocampus of these animals^38^. In this study, there was a trend of suppressed [^11^C]PBB3-positive tau deposition in the brainstem of FTLD-tau mice at 24 weeks after tau-v injection, but the difference in PET signals between the treatment groups was not statistically significant (Supplementary Fig. 2c, d).

### Tau vaccine ameliorated tau pathology of FTLD-tau mice

**Fig. 3:**
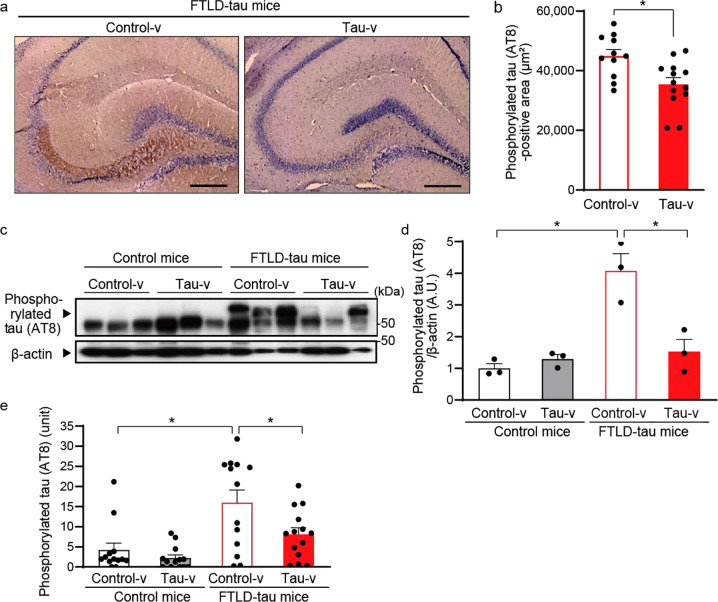
Amelioration of tau pathology and neuroinflammation by nasal vaccination. **a** A representative image of the accumulation of phosphorylated tau detected by anti-phosphorylated tau antibody (AT8, phosphorylation sites Ser202 and Thr205) in the hippocampus of FTLD-tau mice at 24 weeks after vaccination. Immunostaining of the hippocampal CA3 sector revealed attenuation of AT8 immunoreactivity in FTLD-tau mice as a consequence of tau-v treatment. Scale bars: 300 µm. **b** Quantification of the phosphorylated tau-positive area of the hippocampal CA3 sector detected by anti-phosphorylated tau antibody (AT8, phosphorylation sites Ser202 and Thr205). Tau-v treatment decreased the phosphorylated tau-positive area. *p < 0.001 by *t*-test (control-v-treated FTLD-tau mice, *n* = 11; tau-v-treated FTLD-tau mice, *n* = 13). **c** Western blot analysis of the phosphorylated tau accumulation detected by anti-phosphorylated tau antibody (AT8). **d** Quantification of the phosphorylated tau accumulation detected by anti-phosphorylated tau antibody (AT8) in western blot analysis. Tau-v-induced suppression of phosphorylated tau deposition in the FTLD-tau mouse hippocampus was demonstrated by immunoblotting total homogenates [*F* (3,8) = 16.475, *p* < 0.005] by ANOVA. *p < 0.005 by Bonferroni’s post-hoc test (*n* = 3 mice in each group). **e** ELISA indicated an increase in phosphorylated tau detected by anti-phosphorylated tau antibody (AT8) in the hippocampus of FTLD-tau mice. The phosphorylation level was partially attenuated by tau-v treatment [*F*(1,16) = 9.3421, *p* < 0.05] by ANOVA. *p < 0.05 by Bonferroni’s post-hoc test (*n* = 13–14 mice in each group). All blots were derived from the same experiment and were processed in parallel.

The decreased deposition of phosphorylated tau in hippocampal CA3 neurons of FTLD-tau mice by tau immunization was further proven by immunohistochemical assays of postmortem mouse brain tissues collected at the predetermined endpoint of the therapeutic evaluation at 9 months of age (Fig. 3a, b). Reduced hyper-phosphorylated tau levels in tau-v-treated mice were confirmed by western blot analysis of brain extracts with phosphorylated tau-specific antibody (Fig. 3c, d). We then carried out an enzyme-linked immunosorbent assay (ELISA) measurement of tau proteins extractable from FTLD-tau mouse brain samples by reassembly buffer with high salt contents (RAB-HS)^30^ and found a significant decrease in the amount of phosphorylated tau by tau-v treatment (Fig. 3e). Immunohistochemistry for ionized calcium-binding adapter molecule 1 (Iba-1) indicated the suppression of microgliosis as a consequence of the tau-v vaccination (Supplementary Fig. 3). This change was accompanied by a decrease in the TSPO-positive microglia in agreement with the TSPO-PET data, and a decrease of glial fibrillary acidic protein (GFAP) expression, which indicated the suppression of astrocytosis by tau-v in the hippocampus of FTLD-tau mice (Supplementary Fig. 3). Immunization with tau-v did not provoke the infiltration of CD3e-positive T lymphocytes in the brains of mice (Supplementary Fig. 3).

### Tau vaccine improved cognitive impairment of FTLD-tau mice

**Fig. 4:**
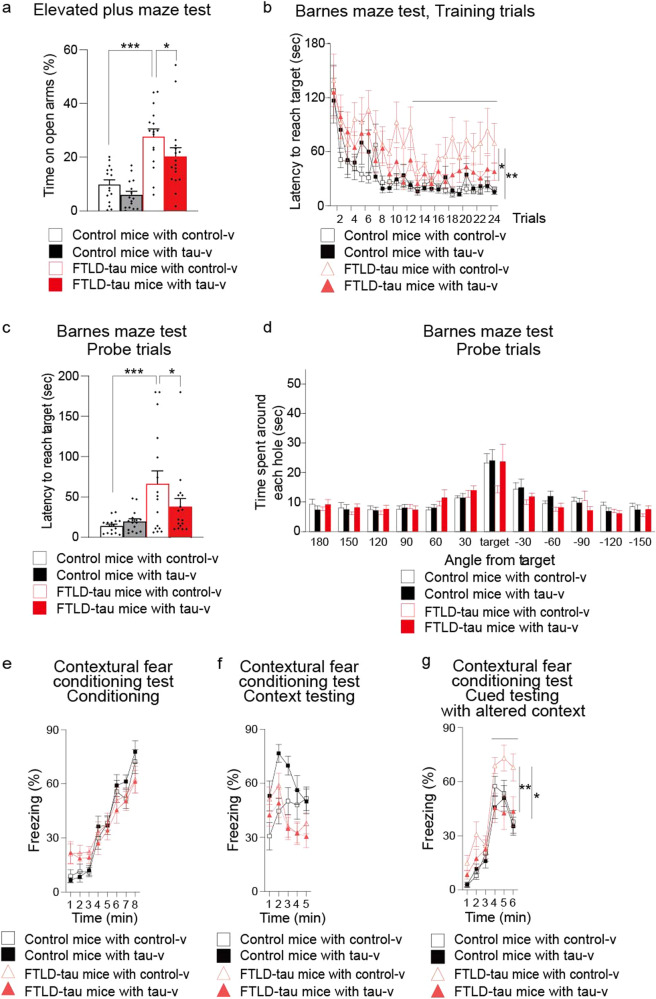
Improvement of cognition by nasal vaccination. **a** Elevated plus maze test. Time spent on open arms was significantly increased in FTLD-tau mice treated with control-v, and this change was suppressed by tau-v treatment. One control-v-treated control mouse and one tau-v-treated control mouse dropped from the open arms, thus failing to complete the task [*F*(3, 59) = 15.461, *p* < 0.0001, control/control-v vs. FTLD-tau/control-v: *p* < 0.0001, FTLD-tau/control-v vs. FTLD-tau/tau-v: *p* = 0.0379]. Tested by one-way ANOVA, followed by Fisher’s LSD test (*n* = 15–17 mice in each group). **b**–**d** Barnes circular maze test. In the training trials **b**, an increase of latency to reach the target hole in the second half of the trials was observed in control-v-treated FTLD-tau mice, but was reversed by tau-v treatment (*F*(3, 61) = 4.506, *p* = 0.0064, control/control-v vs. FTLD-tau/control-v: *p* = 0.0064, FTLD-tau/control-v vs. FTLD-tau/tau-v: *p* = 0.0385). Tested with repeated measures ANOVA. In the probe trials **c** 24 h after the last (24th) training trial, tau-v also significantly attenuated the elongation of latency to reach the target hole in FTLD-tau mice (*F*(3, 61) = 5.877, *p* = 0.0014, control/control-v vs. FTLD-tau/control-v: *p* = 0.0003, FTLD-tau/control-v vs. FTLD-tau/tau-v: *p* = 0.0408). Tested with one-way ANOVA followed by Fisher’s LSD test. The decrease in time spent around the target hole by FTLD-tau mice **d** was significantly suppressed by tau-v treatment (*p = 0.0867). Time spent around the target versus the next holes (mean duration) was tested by paired *t-*test (*n* = 16–17 mice in each group). **e**–**g** Fear conditioning test. Freezing times (expressed as percent of total time) in each minute of conditioning **e**, context testing **f**, and cued testing **g** with altered context were analyzed. No significant abnormalities in FTLD-tau mice compared to control mice were observed in conditioning and context testing **e**, **f**. However, freezing time with an auditory cue was significantly increased in control-v-treated FTLD-tau mice, but was reversed to control level by tau-v treatment **g** (*F*(3, 61) = 3.887, *p* = 0.00131, control/control-v vs. FTLD-tau/control-v: *p* = 0.0257, FTLD-tau/control-v vs. FTLD-tau/tau-v: *p* = 0.0042). Tested with repeated measures ANOVA followed by Fisher’s LSD test (*n* = 16–17 mice in each group). All blots were derived from the same experiment and were processed in parallel.

Finally, we conducted an array of behavioral test batteries for the treated mice at 7–9 months of age (Fig. 4, Supplementary Fig. 4; the test data are summarized in Table 1). No difference in body weight and no deficits in motor and sensory functions were detected among the tested animals grouped by genotypes and treatments (Supplementary Fig. 4a–d, and Table 1). By contrast, control-v-treated FTLD-tau mice exhibited a loss of hazard perception in the elevated plus maze test (Fig. 4a and Supplementary Video 1) and disturbed spatial memory in the Barnes maze test (Fig. 4b–d), but both were significantly alleviated by treatment with tau-v. Impairments of contextual memory and/or hazard perception as monitored by the fear conditioning test were also observed in control-v-treated FTLD-tau mice, but again were ameliorated by the tau-v vaccination (Fig. 4e–g). These cognitive impairment phenotypes are known to be associated with the hippocampus and other limbic areas, reinforcing the notion that tau-v vaccine serves to rescue hippocampal neurons from tau-triggered dysfunctions. Other behavioral scores, such as performances in the open field (Supplementary Fig. 4e–h), social interaction (Supplementary Fig. 4i–m), rotarod treadmill (Supplementary Fig. 4n), hot plate (Supplementary Fig. 4o), prepulse inhibition (Supplementary Fig. 4p and q), and tail suspension (Supplementary Fig. 4r) tests were not altered in FTLD-tau mice relative to control mice irrespective of the treatment. Taken together, these findings provided evidence for the repression of tau-induced cognitive deteriorations in vaccinated FTLD-tau mice.

**Table 1:** Summary of behavior analysis.

Tests	FTLD-tau/control-v vs. control/control-v	FTLD-tau/tau-v vs. FTLD-tau/control-v	Related behavioral abnormalities	Assessed regions	Figures
Elevated plus maze test	Less hazard perceptive (*p* < 0.0001)	Improved (*p* = 0.0379)	Anxiety, hazard perception	Cognitive system (hippocampus, cingulate cortex)	Fig. 4a
Barnes maze test	Memory loss (*p* = 0.0064)	Improved (*p* = 0.0385)	Spatial working memory, reference memory	Cognitive system (hippocampus)	Fig. 4b–d
Contextual fear conditioning test	Less hazard perceptive (*p* = 0.0257)	Improved (*p* = 0.0042)	Contextual memory, hazard perception	Cognitive system (hippocampus, amygdala)	Fig. 4e–g
Body weight	Decreased (*p* = 0.0233)	No change	–	–	Suppl. Fig. 4a
Body temperature	Tendency to decrease	Recovered (*p* = 0.0119)	–	–	Suppl. Fig. 4b
Grip strength	No change	No change	Muscle strength	Motor system	Suppl. Fig. 4c
Wire hang test	No change	No change	Muscle strength	Motor system	Suppl. Fig. 4d
Open field test	No change	No change	Anxiety, exploratory locomotion	Prefrontal cortex, cingulate cortex	Suppl. Fig. 4e–h
Social interaction test	No change	No change	Anxiety in novel situation, sociality	Cognitive system (hippocampus, cingulate cortex)	Suppl. Fig. 4i–m
Rotarod treadmill test	Hyperactive (*p* = 0.014)	No change	Muscle weakness, motor activity, coordination	Motor tract, cerebellum	Suppl. Fig. 4n
Hot plate test	No change	No change	Pain and temperature sensation	Supraspinal sensory tract	Suppl. Fig. 4o
Prepulse inhibition test	No change	No change	Sensorimotor gating, startle response, hearing acuity	Prefrontal cortex, hippocampus, striatum	Suppl. Fig. 4p, q
Tail suspension test	No change	No change	Behavioral despair	Stria terminalis (basal forebrain)	Suppl. Fig. 4r
Righting reflex	No change	No change	Postural maintenance, muscle strength	Vestibular system, muscle	Not shown
Whisker twitch	No change	No change	Tactile sensation	Sensory system	Not shown
Ear twitch	No change	No change	Tactile sensation	Sensory system	Not shown
Reaching	No change	No change	Visual acuity	Visual system	Not shown
Key jangling	No change	No change	Hearing acuity	Auditory system	Not shown

Tests are in ascending order based on size of the rescue effect.

## Discussion

We developed a nasal anti-tau vaccination using a SeV vector carrying a secreted form of human tau with P301S mutation as an immunogen. The vaccination improved tau pathologies and associated histopathological abnormalities in FTLD-tau mice, including a reduced accumulation of phosphorylated tau proteins and neuroinflammation. Suppression of the neuronal loss and detrimental microgliosis in these mice by the tau-v treatment was also demonstrated by volumetric MRI and TSPO-PET, respectively. Moreover, improved cognitive performance of FTLD-tau mice by nasal vaccination was observed in multiple behavioral tasks.

Since the three-dimensional structure of misfolded tau presents complexity, and it takes on a more complex structure by binding to various proteins and lipids^39,40^, antibodies against naïve protein caused by vaccination may be more effective. However, given the high cost of many antigen production systems, nasal vaccination offers a distinct advantage over other routes.

Phosphorylated tau and its assemblies have been mechanistically implicated in diverse tauopathies as pathological culprits, and studies on different lines of tau transgenic mice have indicated crucial roles played by soluble but misfolded tau species comprised of monomers and oligomers with pathological conformations and posttranslational modifications in neurodegenerative pathogenesis^30,41,42^. The present observations have provided evidence for the therapeutic potency of a viral vector-assisted active immunization directed against these non-fibrillar tau proteins, as well as for its safe properties without marked emergences of deleterious microgliosis and T-lymphocyte^43^ meningoencephalitis. These features may offer a new approach that mimics and targets ‘natural’ tau released from affected neurons and is conceptually distinct from previously documented methods using synthetic peptidic fragments of tau proteins as immunogens^44–46^. The reactivity of the immunized mouse plasma with tau lesions in FTLD-tau mouse brains also supports the efficient production of autoantibodies against tau proteins with pathological conformations. This production could be facilitated by the incorporation of P301S mutation in the SeV vector-encoded sequence, which is likely to augment the propensity of the secreted tau to the formation of anomalous conformers. Given that these pathologic conformations are commonly shared by various tauopathies, the current nasal vaccine would be efficacious in treating AD and different FTLD-tau subtypes. In addition to the consensus view that normal tau promotes the assembly of tubulins and stabilizes microtubules in axons, there has emerged a notion that misfolded tau is one of the non-prion amyloids spreading via cell-to-cell transmission^46–48^. The secretion of tau from live neurons was also revealed by a microdialysis of mice^49^. In this consideration, antibodies resulting from immunization with extracellular tau molecules produced by tau-v vaccination could potently target transmitting tau proteins and/or render microglia reactive with these pathogenic molecules.

This study has successfully demonstrated the efficacy of a nasal vaccine, but the precise mechanism for the efficacy is not clear, and no specific antibody was identified. However, we validated the findings about effectiveness with multiple behavioral tasks and imaging using MRI and PET. Future work should consider the mechanism of the vaccine’s effectiveness.

Besides therapeutic efficacies, it is of practical significance that the treatment with tau-v did not worsen noticeable neuroinflammation. According to the present longitudinal PET data, tau-v may transiently trigger the activation of TSPO-positive microglia, but the vaccination thereafter diminished deleterious gliosis associated with tau pathologies and neurodegeneration. The magnitude of the inflammatory reactions could be determined by multiple factors, including modes of immunizations, since repeated vaccinations with phosphorylated tau peptides emulsified in an adjuvant were reported to elicit neuroinflammation^16^. Although the safety of SeV vectors has been demonstrated in humans^27–29^, unexpected autoimmune responses and possible secondary effects of vaccinations with tau protein need to be taken into account. Previous reports of tau vaccinations using a short form of phosphorylated tau proteins^50^ and a cysteinated tau peptide containing structural determinants^51^ showed safety with efficacy in rodents, and they are currently undergoing clinical trials. Our tau vaccine also did not demonstrate any overt adverse actions in a mouse model; however, careful consideration will be needed to evaluate long-term safety.

To conclude, we propose nasal vaccinations with SeV vector as a useful immunotherapeutic approach to neurodegenerative tauopathies for a broad range of the population in our aging society.

## Methods

### Ethics statement

All animals were cared for and procedures performed in accordance with the Institutional Guidelines for the care and use of laboratory animals in Kyoto University and National Institute of Radiological Science (NIRS), and all experiments were approved by the Animal Experiment Committees of Kyoto University and NIRS.

### SeV vector

SeV vector deleted fusion gene (SeV/ΔF) to eliminate transmission was obtained from ID Pharma (Tsukuba, Japan). SeV vector carrying the cDNA of human tau isoform with one N-terminal 1N4R with the P301S mutation fused to a signal sequence for directing gene products to a secretory pathway was constructed. The signal sequence was obtained from the cDNA of the N-terminal in APP. SeV vector expressing EGFP (SeV-EGFP) for use as a gene-expressing control was also constructed to check the gene expression induced by SeV vector in nasal mucosa. The nasal cavity was removed one week after the infection of SeV-EGFP, fixed in 4% formaldehyde at room temperature for 2 h, decalcified by 0.24 M EDTA solution at room temperature for 5 days, and transferred into 30% sucrose overnight. The tissue was then embedded in Tissue Mount (Chiba Medical, Chiba, Japan), sectioned at 10-μm thickness by a microtome, and observed by fluorescence microscopy.

To confirm the secreted tau protein expression, we transfected SeV vector carrying human P301S (1N4R) tau protein gene with a secretion signal to HEK293T cells. Transfected tau gene expression was confirmed by immunoprecipitation.

### Immunoblotting

Tau protein levels were determined by homogenizing brains in 2 ml/g tissue of ice-cold high-salt reassembly buffer (RAB-HS) (0.1 M MES, 1 mM EGTA, 0.5 mM MgSO_4_, 0.75 M NaCl, 0.02 M NaF, 1 mM PMSF) and protease inhibitor cocktail (Roche Diagnostics, Basel, Switzerland), followed by centrifugation at 50,000×*g* for 40 min at 4 °C in a Beckman TLA-55 ultracentrifuge. Protein concentrations were determined, and sodium dodecyl sulfate–polyacrylamide gel electrophoresis (SDS–PAGE) followed by western blot analysis was performed as described^52,53^. The following primary antibodies were used: phosphorylated tau (AT8) (1:1,000, #. MN1020, Thermo Scientific, Waltham, MA) and β-actin (1:5,000, #. 5441, Sigma). Uncropped images of the original blots were supplied as a supplementary figure (Supplementary Fig. 5).

### Dot blot analysis

HEK293T cells were infected with SeV vector carrying control-v or tau-v. After 24 h, the medium was changed to serum-free medium containing DMEM/F12 (Life Technologies) and Neurobasal (Life Technologies) mixed at a 1:1 ratio, 1% N_2_ supplement (Life Technologies), 2% B27 (Life Technologies), and the cells were cultured for an additional 48 h. The cells were then harvested and lysed in TBS containing protease inhibitor and phosphatase inhibitor. After sonication and centrifugation at 13,000 × *g* for 15 min, each of the lysate samples (1.2 µg/spot) was loaded on a nitrocellulose membrane (0.45 µm pore size, GE Healthcare, Chicago, IL). The cell culture medium was collected and centrifuged at 200 × *g* for 3 min to remove debris. The same amount of supernatant from each sample was concentrated from 500 to 50 µl using Vivaspin (GE Healthcare), and its molecular weight cut-off for the filtration membrane was 10 kDa. 2 µl of each concentrated sample was loaded onto nitrocellulose membranes. Membranes were blocked with 5% skim milk, hybridized with the appropriate antibodies, and visualized using Western Lightning Plus-ECL (PerkinElmer, Inc.). Images were acquired on ImageQuant LAS 4000 (GE Healthcare). The following antibodies were used: mouse monoclonal antibody against misfolded tau (TOC1, gifted by Dr. Binder) and mouse monoclonal antibody against human tau (Tau12, #. MAB2241, Millipore, Burlington, MA).

### Animals

We previously established transgenic mice expressing mutant (P301S) human T34 isoform tau (1N4R) on a B6C3H/F1(C3H) background^30^, and we used a congenic strain created by 10× backcrossing the transgenic mice and wild-type offspring.

### SeV vaccine administration

SeV vectors (5 × 10^7^ cell-infectious units/head) were administered weekly to each 3-month-old mouse intranasally in a 20-µl volume with PBS.

### Tissue-tau immunoreactive antibody assay

P301S brain sections fixed in 4% paraformaldehyde were permeabilized in PBS containing 0.2% Triton X-100 for 10 min at room temperature, followed by blocking for 30 min with 2% BSA and 10% horse serum. The serum of mice 6 months after vaccination was diluted 30×, 100×, 300×, 1,000×, and 3,000×, and then applied to P301S brain sections. After incubation with the diluted serum overnight at 4 °C, the brain sections were washed three times with PBS-T and incubated with anti-mouse IgG secondary antibodies for 1 h at room temperature, followed by incubation with streptavidin–biotin-peroxidase. The maximal dilution of plasma that gave positive staining was estimated as the tangle immunoreactivity titer^54^.

### Measurement of anti-tau antibody titer

Anti-tau antibody titer in brain lysates and serum was evaluated by ELISA assay. The brain samples and serum of FTLD-tau mice were collected 6 months after vaccination. Frozen brain samples were dissolved in TBS buffer containing protease inhibitor (Roche) by homogenization and sonication. Samples were centrifuged at 13,000 × *g* for 15 min at 4 °C, and protein concentrations in the supernatants were determined with bicinchoninic acid (BCA) assay kit (Thermo Fisher Scientific). Total protein extracts of brain lysates were diluted to 500 μg/100 μl. Serum samples were diluted to 1:50. 96-well plates (Greiner) were coated with 1 μg/ml P301S tau recombinant protein in 0.05 M sodium carbonate buffer (pH 9.4) at 4 °C overnight. After washing and blocking with 1% BSA–PBS-T for 1 h, 100 μl of brain lysates or serum was applied to the plates and incubated for 2 h at room temperature. For detection, the plates were incubated with sheep anti-mouse IgG F(ab)’2 fragment linked to a horseradish peroxidase (GE Healthcare) at 1:5,000 dilution for 1 h at room temperature, followed by incubation with tetramethyl benzidine solution (BD) for 15 min. The absorbance at 450 nm was measured by VersaMax (Molecular Device, Sunnyvale, CA).

### Volumetric MRI assays

MRI studies of both control and vaccinated mice were conducted at 24 weeks after the initiation of the vaccine treatment using a 7.0-T MRI scanner (magnet, Kobelco and JASTEC, Kobe and Tokyo Japan; console, Bruker Biospin, Ettlingen, Germany) with a volume coil for transmission (Bruker) and a two-channel phased-array coil for reception (Rapid Biomedical, Wuerzburg, Germany). Rectal temperature was continuously monitored by an optical fiber thermometer (FOT-M, FISO, Canada) and maintained at 36.5 ± 0.5 °C using a heating pad (Rapid Biomedical) and warm air. The first imaging slices were carefully set at the rhinal fissure with reference to the mouse brain atlas. Signal excitation and detection were performed by a 25-mm resonator. T2-weighted 3D spin-echo rapid acquisition with relaxation enhancement (RARE) MRI scans were performed. The imaging parameters were as follows: TR/effective TE = 4200/36 ms, Fat-Sup = on, NA = 4, RARE factor = 8, number of slices = 13, and scan time = 6 min 43 s. Frequency selective saturation pulses and crusher magnetic field gradients were used for fat suppression. An anatomical volume of interest (VOI) was manually defined on the hippocampus of the MRI images using PMOD® software (ver 3.6, PMOD Technologies Ltd, Zurich, Switzerland), and VOIs were measured.

### PET imaging

PET scans were performed using a microPET Focus 220 animal scanner (Siemens Medical Solutions, Erlangen, Germany)^55^. Control and vaccinated mice were anesthetized with 1.5% (v/v) isoflurane, and a 30-G needle connected to a 1-ml polypropylene syringe via a length of polyethylene tubing was inserted into the tail vein. After transmission scans for attenuation correction using a 68Ge–68Ga point source, emission scans were acquired for 60 min in 3D list mode with an energy window of 350–750 keV, and the intravenous injection of [^11^C]Ac5216 (13.26 ± 14.27 MBq) or [^11^C]PBB3 (30.28 ± 22.34 MBq) was performed immediately. Summation images from 0 to 60 min after [^11^C]Ac5216 or [^11^C]PBB3 injection were reconstructed with maximum a posteriori reconstruction, and dynamic images were reconstructed with filtered back-projections using a 0.5 mm Hanning filter. To analyze the [^11^C]Ac5216-PET data, VOIs were placed on the hippocampus and striatum using PMOD image analysis software (PMOD Group) with reference to the MRI template at 0 and 3 weeks after initiation of the treatment and to individual MRI data at 24 weeks. Tracer uptake in each VOI was estimated as the percentage of injected dose per tissue volume (%ID/ml). To analyze the [^11^C]PBB3-PET data, VOIs were defined on the brain stem and cerebellum with reference to the MRI template at 24 weeks.

### Immunohistochemistry

Mice were deeply anesthetized and transcardially perfused with 15 ml phosphate-buffered saline (PBS). The brains were removed, immersion-fixed for 24 h with 4% paraformaldehyde in PBS, and then transferred to 20% sucrose solution at 4 °C for at least 4 days. After cryoprotection, the brains were rapidly frozen by heat exchange from vaporized CO_2_ gas (−70 °C), and then sections (12 μm) were cut with a cryostat. The brains were immunostained using streptavidin–biotin-peroxidase^52,53^. The following antibodies were used: mouse monoclonal antibody against phosphorylated tau (AT8, 1:1,000, #. MN1020, Thermo Fisher Scientific), rabbit polyclonal antibody against Iba1 (1:1,000, #. 019-19741, WAKO, Osaka, Japan), goat polyclonal antibody specific for TSPO (1:1,000, #. sc-30920, Santa Cruz, Santa Cruz, CA), rabbit polyclonal antibody specific for GFAP (1:1,000, #. Z033401, DAKO, Santa Clara, CA), and hamster polyclonal antibody specific for CD3e (1:1,000, #. 553058, BD Biosciences, Franklin Lakes, NJ).

### ELISA

Hippocampal human phosphorylated tau protein was evaluated by ELISA assay using anti-phosphorylated antibody (AT8, #. MN1020, Thermo Fisher Scientific). Microtiter plates were coated with 3 µg/ml AT8 antibody in 0.05 M sodium carbonate buffer (pH 9.4) at 4 °C overnight. After washing and blocking with TBS-T containing 1% BSA, 100 µl of diluted mouse hippocampus RAB-HS soluble fractions (the protein concentration of each sample was adjusted) was added, and the incubation was carried out for 2 h at room temperature. Serial dilutions of collected FTLD-tau mouse brain cortical lysates ranging from 1:100 to 1:102,400 were used as positive control. For detection, the plates were incubated with 2 μg/ml rabbit anti-human tau protein antibody (#. A0024, DAKO), followed by sheep anti-rabbit IgG F(ab)′2 fragment linked to horseradish peroxidase (GE Healthcare) at 1:3000 dilution. After incubation with tetramethylbenzidine solution (BD Bioscience) at room temperature for 30 min, absorbance at 450 nm was read on an automated plate reader (Model 353; Thermo Fisher Scientific). The amount of phosphorylated tau protein of positive control was defined as 1000 units, and unknown titers of samples were determined by interpolation from a standard curve generated by positive control standards of known dilution. All samples were analyzed in duplicate.

### Behavioral analysis

For the behavioral analysis, mice were housed 3 or 4 per cage in a room with a 12-h light/dark cycle (lights on at 7:00 a.m.) with access to food and water ad libitum. Behavioral testing was performed between 9:00 a.m. and 6:00 p.m. After the tests, the equipment was cleaned with super hypochlorous water to prevent any bias due to olfactory cues. All behavioral testing procedures were approved by the Animal Care and Use Committee of Kyoto University Graduate School of Medicine.

The neuromuscular strength test was performed with 28-week-old male mice, using both the grip strength test and wire hang test. A grip strength meter (O’Hara & Co., Tokyo, Japan) was used to assess forelimb grip strength. Mice were lifted and held by their tail so that their forepaws could grasp a wire grid. The mice were then gently pulled backward by the tail with their posture parallel to the surface of the table until they released the grid. The peak force applied by the forelimbs was recorded in Newtons (N). Each mouse was tested three times, and the greatest value was used for statistical analysis. In the wire hang test, the mouse was placed on a wire mesh that was then inverted and waved gently, so that the mouse gripped the wire. Latency to fall (in seconds: s) was recorded, with a 60-s cut-off time. Our 28-week-old FTLD-tau mice showed significantly lower body weight, and tended to show lower body temperature, lower grip strength, and longer duration in the wire hang test.

The other tests were performed as previously described^34^. In the elevated plus-maze test in a novel environment (one-chamber social interaction test), a genotypic mismatch was found in two pairs (one FTLD-tau mouse/control-v pair and one FTLD-tau mouse/tau-v pair), and they could not be analyzed.

The applications used for the behavioral studies (Behavioral Image EP, Behavioral Image SI, Behavioral Image BM, and Image FZ) were based on the public domain NIH Image program (developed at the U.S. National Institutes of Health and available on the Internet at http://rsb.info.nih.gov/nih-image/ website) and ImageJ program (http://rsb.info.nih.gov/ij/ website).

### Statistical analysis

Statistical analysis was conducted using StatView (SAS Institute, Cary, NC), Kyplot (Kyens Lab, Tokyo, Japan), and SPSS version 21.0 (IBM SPSS Statics, Chicago, IL). Data were analyzed by Student’s *t*-test, Mann–Whitney’s *U*-test, paired *t*-test, and for multiple comparisons, one-way ANOVA, repeated measures ANOVA, or Kruskal–Wallis test, with successive post hoc analyses. Statistical significance was defined as *p*-value < 0.05. Values in graphs are expressed as means ± SEM.

### Reporting summary

Further information on research design is available in the Nature Research Reporting Summary linked to this article.

## Supplementary information

Supplementary Information

Description of Additional Supplementary Files

Supplementary Movie 1

Supplementary Movie 2

Supplementary Movie 3

Reporting Summary

## Data Availability

The data that support the findings of this study are available from the corresponding author upon reasonable request.
