# Supplementary material for: Nasal vaccine delivery attenuates brain pathology and cognitive impairment in tauopathy model mice
Source: NPJ Vaccines. 2020 Mar 25;5:28. doi: 10.1038/s41541-020-0172-y (PMC7096417; doi:10.1038/s41541-020-0172-y)
Supplement: Supplementary file 1 — Supplementary Information [file 41541_2020_172_MOESM1_ESM.pdf]

Supplementary Information

Title of manuscript:

Nasal vaccine delivery attenuates brain pathology and cognitive impairment in tauopathy model mice

Author list:

Hiroki Takeuchi, Keiko Imamura, Bin Ji , Kayoko Tsukita, Takako Enami, Keizo Takao, Tsuyoshi Miyakawa, Masato Hasegawa, Naruhiko Sahara, Nobuhisa Iwata, Makoto Inoue, Hideo Hara, Takeshi Tabira, Maiko Ono, John Q. Trojanowski, Virginia M.-Y. Lee, Ryosuke Takahashi, Tetsuya Suhara, Makoto Higuchi, Haruhisa Inoue

Supplementary Figures 1-5

Legends of Supplementary Figures 1-5

Legend of Supplementary Movies

Supplementary Figure 1

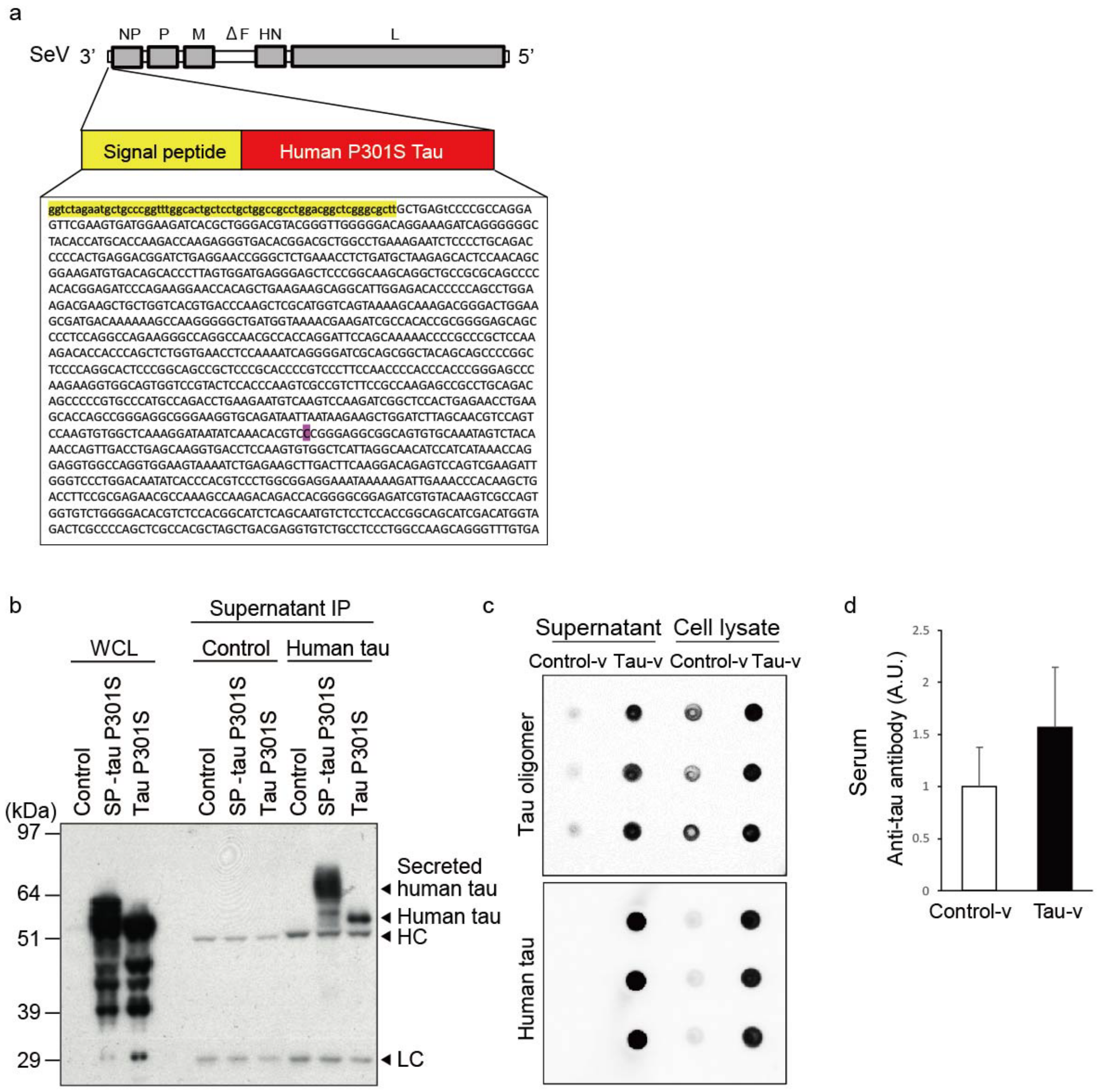

Supplementary Figure 1. Immunogen for vaccination using SeV vector.

(a) Sequence information of the vaccine using SeV vector. Yellow indicates the sequence of the signal peptide. Magenta indicates the mutation of human P301S tau protein (1N4R).

(b) Immunoblot analysis to detect tau proteins secreted from HEK293T cells to the medium. WCL, whole cell lysate; SP-tau P301S, signal sequence combined with P301S mutant tau protein; control vector, SeV vector containing no extrinsic genes; Tau P301S, SeV vector containing P301S mutant tau gene without the signal sequence.

(c) Dot blot analysis shows that tau protein detected by anti-human tau antibody Tau12 and tau oligomer detected by anti-tau oligomer antibody TOC1 were secreted in the culture medium of HEK293T.

(d) Serum anti-tau antibody titer. Tau-v tended to increase the level of serum anti-tau IgG antibody, but not significantly.

Supplementary Figure 2

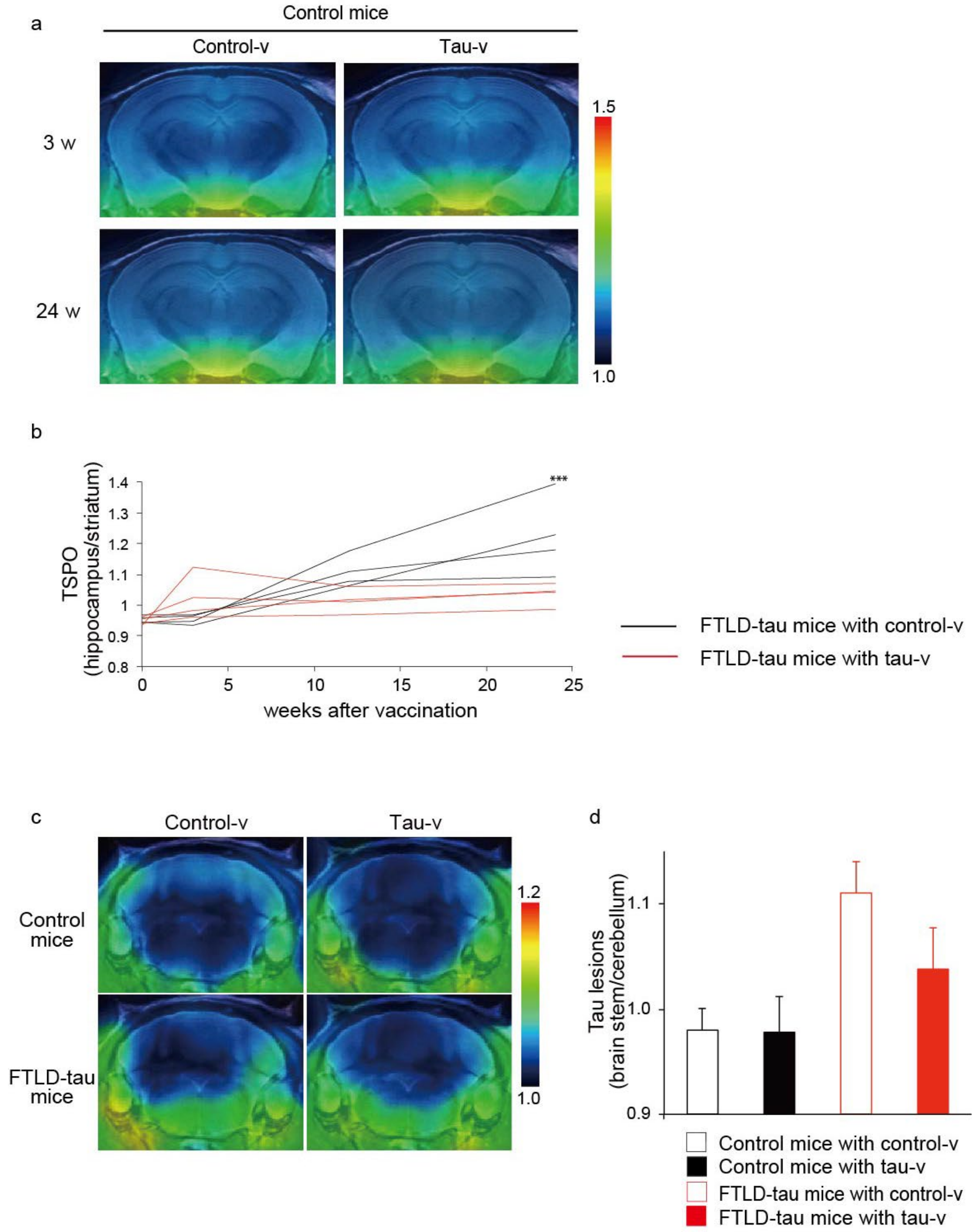

Supplementary Figure 2. Evaluation of vaccination by PET imaging

(a) Coronal TSPO-PET images of non-Tg control mouse brains containing the anterior hippocampus at 3 (top) and 24 (bottom) weeks after vaccination. PET images were generated by averaging the dynamic scan data at 30-60 min after the injection of [^{11}C]Ac5216. The PET images are overlaid on an MRI template. The color scale indicates a ratio of the radioactivity retention to the striatum.

(b) Time course of TSPO changes as quantified by PET (hippocampus-to-striatum ratio of radioactivity at

30-60 min) in each FTLD-tau mouse after treatment with control-v (black lines) or tau-v (red lines). There was a significant main effect of time [$F(3, 18) = 17.66$, $p < 0.0001$] and a significant interaction between time and treatment [$F(3, 18) = 9.034$, $p < 0.001$] by two-way repeated-measures ANOVA. A significant difference between treatment groups at 24 weeks was also observed (***$p < 0.001$ by Bonferroni's post-hoc test, n=3 mice in each group). No statistical significance was observed at 3 weeks.

(c) Coronal tau-PET images of control and FTLD-tau mouse brains including the cerebellum and pons at 24 weeks after treatment (9 months of age). PET images were generated by averaging the dynamic scan data at 30-60 min after the injection of [^{11}C]PBB3. The color scale indicates a ratio of the radioactivity retention to the cerebellum.

(d) Amounts of tau lesions determined by measuring the brain stem-to-striatum ratio of radioactivity at 30-60 min. There was a significant main effect of genotype [$F(1, 12) = 7.076$, $p < 0.05$] by 2-way ANOVA, but no significant effect of treatment (control-v-treated control mice, n=3; tau-v-treated control mice, n=3; control-v-treated FTLD-tau mice, n=5; tau-v-treated FTLD-tau mice, n=5).

Supplementary Figure 3

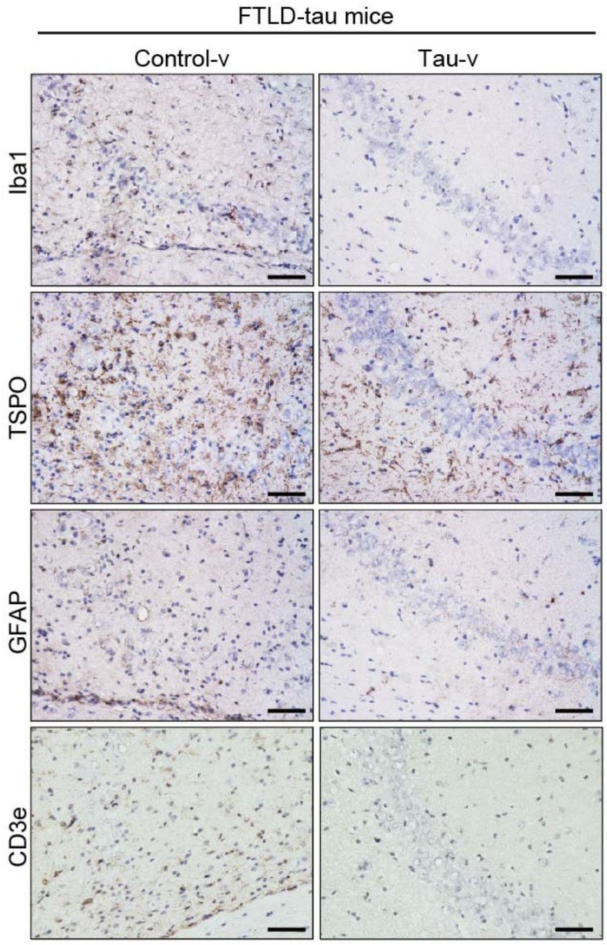

Supplementary Figure 3. Gliotic change and lymphocyte infiltration.

Representative images of Iba-1, TSPO, GFAP and CD3e-positive cells in the hippocampal CA3 sector of FTLD-tau mice. Scale bars: 50 μm. Tau-v treatment indicated a decrease of Iba1-positive cells, TSPO-positive cells, and GFAP-positive cells in FTLD-tau mice without an increase of CD3e-positive cells.

Supplementary Figure 4

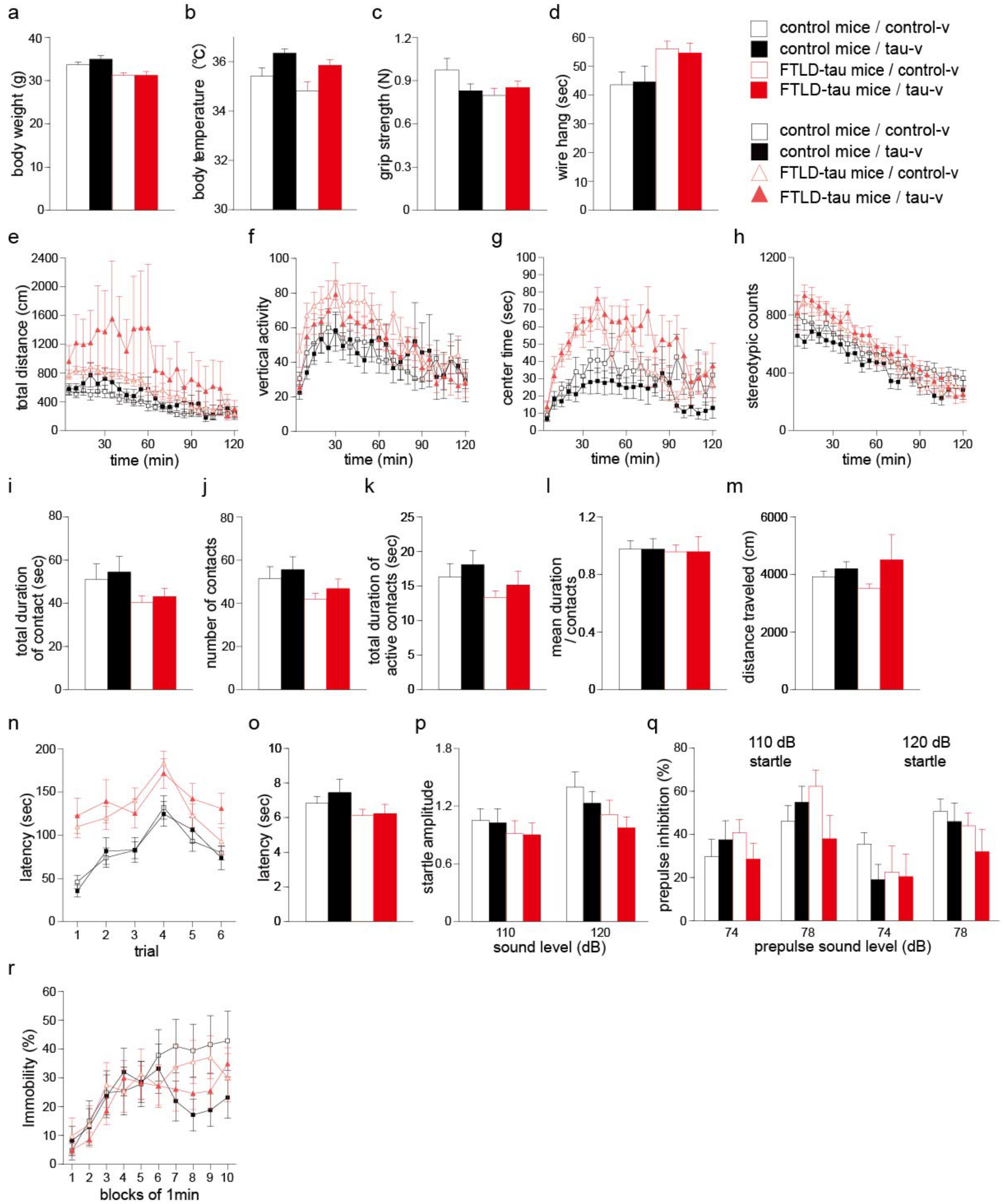

Supplementary Figure 4. Phenotypic assays in mice following vaccination

General condition and sensorimotor abilities of mice. FTLD-tau mice showed significant lower body weight [$F(3, 61) = 6.151$, $p = 0.001$, control mice / control-v vs FTLD-mice / control-v: $p = 0.0233$, control mice / tau-v vs FTLD-tau mice / tau-v: $p = 0.0008$] (a). The tendency of lower body temperature in control-v-treated FTLD-tau mice was reversed by tau-v treatment [$F(3, 61) = 5.154$, $p = 0.0031$, control mice / control-v vs FTLD-tau mice / control-v: $p = 0.1521$, FTLD-tau mice / control-v vs FTLD-tau mice / tau-v: $p = 0.0119$] (b). Grip strength was unchanged [$F(3, 61) = 1.842$, $p = 0.149$] (c), but there was a longer

duration in the wire hang test for FTLD-tau mice [$F(3, 60) = 2.502$, $p = 0.0678$] (d). (E-H) Open field test. No significant main effects of genotype and treatment and no significant interaction between these factors were found in measurements of total distance [$F(3, 61) = 1.268$, $p = 0.2935$] (e) or vertical activity [$F(3, 61) = 1.024$, $p = 0.3886$] (f). The center time was increased in FTLD-tau mice compared to control mice [$F(3, 61) = 6.796$, $p = 0.0005$, control mice / tau-v vs FTLD-tau mice / tau-v: $p < 0.0001$], but no significant effects of tau-v treatment were demonstrated (g). Stereotypic counts showed no significant effects of tau-v treatment either [$F(3, 61) = 2.165$, $p = 0.1015$] (h). The data were examined by repeated-measures ANOVA followed by Fisher's LSD test. (i-m) Social interaction test in a novel environment. No significant main effects of genotype and treatment and no significant interaction between these factors were found on total duration of contact [$F(3, 26) = 1.263$, $p = 0.2118$] (i), number of contacts [$F(3, 26) = 1.361$, $p = 0.2766$] (j), total duration of active contacts [$F(3, 26) = 1.229$, $p = 0.3191$] (k), mean duration per contact [$F(3, 26) = 0.019$, $p = 0.9962$] (l) and distance traveled [$F(3, 26) = 0.833$, $p = 0.4878$] (m). Data were examined by one-way ANOVA followed by Fisher's LSD test. A hyperactive performance in rotarod test [$F(3, 61) = 10.84$, $p < 0.0001$, control / control-v vs FTLD-tau / control-v: $p = 0.014$] (n) was observed in FTLD-tau mice relative compared to control mice, and tau-v treatment did not affect this phenotype. There were no significant main effects of genotype and treatment and no significant interaction between these factors on latency in the hot plate test [$F(3, 61) = 1.253$, $p = 0.299$] (o). (p,q) Startle response/prepulse inhibition test. There were no significant main effects of genotype and treatment and no significant interaction between these factors on startle amplitude at 110 dB [$F(3, 61) = 0.341$, $p = 0.796$] and 120 dB [$F(3, 61) = 1.751$, $p = 0.166$] (p), on prepulse inhibition at 110 dB startle with 74 dB [$F(3, 61) = 0.663$, $p = 0.578$] and 78 dB [$F(3, 61) = 1.599$, $p = 0.199$] prepulse sounds, and at 120 dB startle with 74 dB [$F(3, 61) = 0.684$, $p = 0.566$] and 78 dB [$F(3, 61) = 1.036$, $p = 0.383$] prepulse sounds (q). Data were examined by one-way ANOVA. (r) Tail suspension test. No statistical significance was observed for main effects of genotype and treatment and no significant interaction between these factors on immobility time (expressed as percent of total observation time) [$F(3, 61) = 0.063$, $p = 0.578$ by repeated-measures ANOVA] (n=16-17 mice in each group).

Supplementary Figure 5

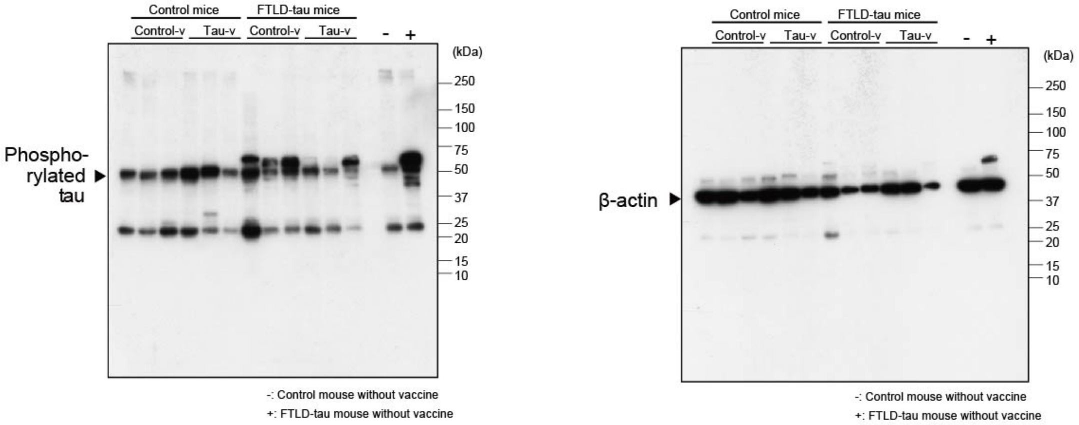

Supplementary Figure 5. Full-length pictures of blots presented in the main figures

Full-length picture of the blots presented in Figure 3c.

Supplementary Movies.

Supplementary Movie 1. Elevated plus maze test of control mouse with control_v

Supplementary Movie 2. Elevated plus maze test of FTLD_tau mouse with control_v

Supplementary Movie 3. Elevated plus maze test of FTLD_tau mouse with tau_v

Legend of the movies:

Representative movies acquired during the elevated-plus maze test. Vertical and horizontal arms are closed and open arms, respectively. Although FTLD-tau mice with control-v spent more time on open arms than the other mice, FTLD-tau mice with tau-v spent more time on closed arms than with control-v.
