# Supplementary material for: Nasal vaccine delivery attenuates brain pathology and cognitive impairment in tauopathy model mice
Source: NPJ Vaccines. 2020 Mar 25;5:28. doi: 10.1038/s41541-020-0172-y (PMC7096417; doi:10.1038/s41541-020-0172-y)
Supplement: Supplementary file 2 — Description of Additional Supplementary Files [file 41541_2020_172_MOESM2_ESM.docx]

Description of Additional Supplementary Files

File Name: Supplementary Movie 1

Description: Elevated plus maze test of control mouse with control_v

File Name: Supplementary Movie 2

Description: Elevated plus maze test of FTLD_tau mouse with control_v

File Name: Supplementary Movie 3

Description: Elevated plus maze test of FTLD_tau mouse with tau_v
